# The Reactive Oxygen Species Scavenger N-Acetyl-L-Cysteine Reduces Storage-Dependent Decline in Integrin *α*_IIb_*β*_3_-Mediated Platelet Function, Inhibiting Pre-Activation of Integrin and Its *β*_3_ Subunit Cleavage

**DOI:** 10.1155/omcl/7499648

**Published:** 2025-04-21

**Authors:** Ehteramolsadat Hosseini, Zahra Beyranvand, Simone M. Schoenwaelder, Fateme Farhid, Mehran Ghasemzadeh

**Affiliations:** ^1^Blood Transfusion Research Center, High Institute for Research and Education in Transfusion Medicine, Tehran, Iran; ^2^Charles Perkins Centre, The University of Sydney, Camperdown, New South Wales, Australia; ^3^Heart Research Institute, Newtown, New South Wales, Australia

**Keywords:** adhesion, integrin *α*_IIb_*β*_3_, N-acetyl-L-cysteine (NAC), platelet, reactive oxygen species (ROS), thrombosis, transfusion, VAS2870

## Abstract

**Background:** Premature activation of integrin *α*_IIb_*β*_3_ plays a central role in the induction and development of the platelet storage lesion (PSL) characterized by an exhausted platelet phenotype that affects adhesion and spreading on fibrinogen. Given the role of reactive oxygen species (ROS) in regulating platelet activation per se, we investigated the effects of a ROS scavenger on reducing the functional decline of platelet integrin *α*_IIb_*β*_3_ during storage.

**Methods:** Platelet-rich plasma-platelet concentrates (PRP-PCs) were either treated with ROS-reducing agents (1 mM N-acetyl-L-cysteine [NAC] or 30 μM NADPH oxidase [NOX] inhibitor, VAS2870) or kept untreated during storage. CD41/CD61 (total integrin *α*_IIb_*β*_3_) expression and PAC-1 binding (specific to active integrin *α*_IIb_*β*_3_ conformation) were analyzed by flow cytometry over a 5 day storage period. Molecular changes in integrin *β*_3_ subunit were evaluated by western blotting. Platelet adhesion/spreading to fibrinogen in the presence of ROS inhibitors was also investigated during storage using fluorescence microscopy.

**Results:** A decrease in the molecular weight of integrin *β*_3_ subunit was observed during platelet storage, and was significantly reduced by NAC but not VAS2870, suggesting proteolytic cleavage of *β*_3_ during storage. Further to this, ROS inhibitors decreased integrin activation and increased platelet adhesion to fibrinogen from day 3 of storage, while NAC but not VAS2870 improved platelet spreading.

**Conclusion:** This is the first report of increasing *β*_3_ cleavage of integrin during storage that was inversely correlated with integrin *α*_IIb_*β*_3_-mediated platelet function. In this regard, as a generic ROS scavenger, NAC was shown to reduce defects in platelet spreading through inhibition of *β*_3_ cleavage. This is in contrast to VAS2870 which selectively inhibits cytosolic NOX alone, suggesting that the reduced platelet function observed during storage may be due to cumulative effects of mitochondrial ROS. Taken together, these studies suggest that adding NAC to platelets may significantly preserve optimal integrin *α*_IIb_*β*_3_ and platelet function during storage. Moreover, as a reversible scavenger, its inhibitory effect can be readily compensated after transfusion.

**Summary**

• Platelet storage was associated with increasing levels of *β*_3_ cleavage of integrin.
• Generic reactive oxygen species (ROS) scavenger, N-acetyl-L-cysteine (NAC) attenuated stored-dependent *β*_3_ cleavage of integrin.
• *β*_3_ cleavage of integrin was inversely correlated with *α*_IIb_*β*_3_-mediated platelet function during storage.
• Reducing state increased platelet adhesion to fibrinogen from day 3 of storage.
• In this regard, NAC but not VAS2870 improved platelet spreading during storage.

## 1. Introduction

Regardless of the methods applied to produce therapeutic platelets, their preparation and transfusion are complicated by time-dependent deleterious alterations, so-called platelet storage lesion (PSL) [1]. Storage-dependent changes in platelet function generally begin with reversible proaggregatory events that inevitably progress to irreversible alterations, including granule release manifested by the expression of proinflammatory molecules, shedding of platelet adhesive receptors, as well as the increase in procoagulant function mainly associated with mitochondrial dysfunction, all of which are orchestrated by platelet metabolic arrest and reactive oxygen species (ROS) generation during storage [2]. These alterations and the need to store platelet products at room temperature make them vulnerable to the risk of bacterial contamination. As such, stored platelet concentrates have a limited shelf life of 5 days, albeit, under certain conditions, storage time can be extended up to 7 days [3].

Subtle pre-activation changes in platelets manifested by shape change and a mild aggregation phenotype may occur during the preparation of platelet products, yet are reversible when allowed to rest [4]. Experimentally, treatment of platelets with a weak agonist such as ADP can mimic this condition in vitro due to the reversible nature of integrin priming [5]. However, in the presence of strong agonists such as collagen, platelets experience stronger stimulatory signals resulting in granule release and irreversible integrin activation, a condition that appears during long-term storage of platelets, especially associated with the presence of small aggregates in platelet bags [6].

Similarly, storage-dependent platelet activation results in the release of soluble agonists that enhance inside out signaling and subsequent initial calcium influx, leading to conformational changes in resting *α*_IIb_*β*_3_ integrin and an increased affinity to soluble fibrinogen [7, 8]. As the main player in platelet function, the activation of *α*_IIb_*β*_3_ integrin has a tightly regulated mechanism. This process is initially triggered by inside out signaling events that enhance integrin's affinity to its primary ligands by modifying the cytoplasmic tail of *β*_3_ through its interaction with an unmasked talin [7]. These modifications propagate to the extracellular domain of the receptor resulting in alteration in the conformation of its ligand binding site from a cryptic to an exposed formation, enabling this receptor to bind to its specific ligands mainly fibrinogen [9–11]. Subsequently, upon the engagement of integrins, their cross-linking through the binding to multimeric ligands (e.g., fibrinogen and Von Willebrand factor [vWF]) induces outside in signaling which triggers the clustering of integrins within the plane of the plasma membrane [12]. This process enhances substrate binding avidity and signaling capacity [13] leading to the irreversible phase of platelet activation which covers its fundamental functions including platelet degranulation, oxidant production, aggregation, adhesion, and spreading [7, 12, 14, 15]. Integrin activation is also associated with an influx in ROS generation due to the enhancement of cytosolic NADPH oxidase (NOX) activity which in interplay with mitochondrial ROS production further enhances the platelet activation state [16, 17].

However, as platelets become more activated, increased outside in signals associated with sustained calcium influx lead to the formation of highly activated procoagulant platelets [14, 18, 19]. Here, in a negative feedback, the sustained elevated intracellular Ca^2+^ concentration can also activate calpain, a member of intracellular cysteine endopeptidases [20, 21], that cleaves the NXXY motif as the most important hot spot located in the *β*_3_ cytoplasmic tail of *α*_IIb_*β*_3_ integrin, which is mainly involved in signal transduction and cytoskeletal–integrin interaction [22].Given the pivotal role of these motifs in integrin function, any condition that increases their removal by active calpain can cause dysfunction of *α*_IIb_*β*_3_ integrin and impaired platelet activity [22, 23], including its adhesion and spreading to the fibrinogen matrix [24]. This may also be relevant during the storage of platelets, especially for longer stored products that are not only affected by such an activation-based mechanism but also suffer from stored-dependent metabolic arrest due to a gradual decrease of consumable ingredients of additive solution in the bags. This is a situation in which, without changing integrin expression during storage, PSL disrupts its function and causes platelet dysfunction [7]. In this context, several lines of evidence also demonstrated that the accumulation of intra-platelet ROS is associated with PSL [6–8], where exaggerated ROS generation triggers deleterious changes, including a disintegrin and metalloproteinase domain-containing protein (ADAM)-dependent receptor shedding [25], calpain-mediated cytoskeletal alteration [26], metabolic changes, and dramatic energy inadequacy due to mitochondrial damages [27]. However, it should be noted that ROS levels generally reflect the state of platelet activity, where under physiological conditions they interfere with the inside out signaling pathways that support integrin activation and platelet function [28, 29]. In this way, once platelets are stimulated, the activation of NOX increases the levels of intra-platelet ROS [28, 30] which can trigger the activation of *α*_IIb_*β*_3_ integrin through Ca^2+^-dependent and -independent pathways [16, 31]. Accordingly, the unwanted stimulation of platelets during storage, together with the gradual increase in ROS production, could further engage integrin with pre-activation and reduce its potential for proper function at later stages, where the platelets also endure metabolic arrest due to their premature activation. This is what was demonstrated by our previous studies in which stored platelets gradually lost their adhesion and spreading ability to the fibrinogen matrix, where their integrin was pre-activated in the presence of increasing levels of ROS during storage [17, 32].

Given the involvement of ROS in platelet activation and their pivotal role in the initiation and development of PSL, and the evidence that inhibition of ROS reduces platelet aggregation or integrin activation [33–35], the current study aims to investigate whether a ROS scavenger or inhibition of NOX as the main source of ROS generation, can attenuate storage-dependent integrin pre-activation, and restore platelet adhesive capacities on the fibrinogen matrix. To this end, we used VAS2870, a specific NOX inhibitor and N-acetyl-L-cysteine (NAC) as a general ROS scavenger, to investigate their effects on the expression pattern of *α*_IIb_*β*_3_ integrin and platelet adhesion/spreading on the fibrinogen matrix during storage. In addition, this study attempted for the first time to investigate the storage-dependent cleavage of the integrin *β*_3_ chain under normal storage conditions in comparison with the reduced state.

## 2. Materials and Method

### 2.1. Reagents

Mouse IgG1, *κ* Isotype controls (PE and FITC conjugated) were from Miltenyi Biotec (Germany). Mouse anti-human CD41 (FITC conjugated), mouse anti-human CD61 (PE conjugated), and PAC-1 (mouse monoclonal antibody against active GPIIb/IIIa conformation, FITC conjugated) were from BD biosciences (USA). Monoclonal antibodies Integrin *β*_3_/ITGB3/CD61 and GAPDH were from Santa Cruz Biotechnology (USA). Fibrinogen, NAC, VAS2870 (3-benzyl-7-(2-benzoxazolyl)thio-1,2,3-triazolo[4,5-d]pyrimidine) and other reagents and chemicals were from Sigma-Aldrich (USA).

### 2.2. Sample Preparation

Samples were collected from eligible volunteer donors at the Iranian Blood Transfusion Organization (IBTO) after obtaining informed consent. For each run of experimental assays, two platelet-rich plasma-platelet concentrates (PRP-PCs) bags were randomly selected and then prepared after passing the release process under IBTO screening regulations. Each bag contained ~ 70 mL of PCs with at least 1 × 10^9^ PLT/mL. For each run of experimental assays, two ABO- and D-matched PRP-PC units were pooled in a closed system using a connecting device instrument (TSCD-II, Terumo Sterile Tubing Welder, Japan) and split into three bags, with their volumes equally adjusted using a digital balance (Sartorius, Germany). The three satellite bags were labeled as control, NAC (1 mM)- or VAS2870 (30 μM)-treated platelets. Under sterile conditions, designated amounts of NAC or VAS2870 dissolved in buffer were added to the indicated bags, while an equal amount of this buffer without either drug was added to the control bag. All bags were then kept at 20°C–24°C in a shaker incubator until the time of sample preparation, when 5 mL of products was taken from each bag via the cord for the required analysis on days 0, 1, 3, and 5 of storage (as well as seven for western blot), under sterile conditions.

### 2.3. Experimental Procedure

#### 2.3.1. Platelet Counting and pH Measurement

For QC assessment as well as sample adjustment, platelet, and WBC count were measured using a hematology full blood analyzer (XE-2100, Sysmex, Milton Keynes, UK) on PCs after preparation and during storage at each time point. pH was also measured at 22°C using a pH meter (826 pH mobile/827 pH lab, Metrohm AG, Switzerland).

#### 2.3.2. Bacterial Cultures

According to the QC protocol, to confirm that PCs were not contaminated during storage; bacterial cultures (including both aerobic and anaerobic cultures) were performed on day 7 for each bag of platelets.

#### 2.3.3. Static Platelet Adhesion

Glass coverslips (12 mm in diameter) were incubated with 100 μg/mL fibrinogen in PBS for 2 h at room temperature, washed, and blocked via incubation with 2% bovine serum albumin for 30 min at room temperature. Excess solution was removed using three washes with Tyrode's buffer and coverslips were kept immersed in Tyrode's buffer until required. Human platelets (2 × 10^7^/mL) were then allowed to adhere and spread on coverslips for 30 min at 37°C. Nonadherent platelets were aspirated and adherent platelets were fixed with 3.7% formaldehyde for 15 min. Adherent platelets were visualized using fluorescence microscopy (100 × objectives). For this purpose, platelets were labeled with DiOC6 before each experiment, and then subjected to adhesion assays. The total number of adherent and percentage spread platelets were calculated. The surface area covered by platelets (μM^2^) was also quantified using ImageJ software (Research Services Branch, National Institute of Mental Health, Bethesda, MD, USA).

#### 2.3.4. Flow Cytometry Analysis

Washed Platelets (2 × 10^7^/mL) were stained with either anti-CD41, -CD61 antibodies to evaluate total *α*_IIb_*β*_3_ expression) or PAC-1 antibody (against the active site of the *α*_IIb_*β*_3_) for 30 min at 37°C (as per manufacturer's instructions). After incubation, platelets were fixed in 1% paraformaldehyde in PBS and subjected to flow cytometry (CyFlow Space, Partec GmbH, Germany) where a total of 20,000 platelet events were acquired. The flow cytometer settings were optimized for the acquisition of platelets by logarithmic signal amplification in all four detectors (forward and side scatter channels and fluorescence channels FL1 and FL2). For analysis, the gate was set around an intact platelet population as defined by forward and side scatter characteristics and confirmed by the presence of platelets expressing CD41. The background staining created by nonspecific binding was detected by IgG-FITC/PE isotype control where the ratios of Geometric mean fluorescence of CD41, CD61, and PAC1 to those of corresponding isotype controls were calculated for each sample. Data were analyzed with FLOWJO software (Tree Star Inc, OR, USA).

#### 2.3.5. Western Blotting

Western blot analysis was used to evaluate the integrin *β*_3_ expression during storage of PRP-PCs. For this purpose, samples obtained from PRP were adjusted to a count of 1 × 10^8^/mL where 1 mL of each sample was then subjected to centrifugation (2000 × *g* for 5 min at 4°C). Platelet pellets were subsequently lysed on ice using lysis buffer containing 100 mM EDTA and Triton 10x in PBS supplemented with a protease inhibitor cocktail. Lysates were then subjected to a second centrifugation step at 7000 × *g* for 10 min at 4°C, and supernatants were collected for further analysis by western blotting. For this purpose, 25 μL from each supernatant was subjected to SDS-PAGE electrophoresis, and protein bands were transferred to the PVDF membrane. After initial blocking and washing steps, the PVDF was immunoblotted in the presence of an integrin *β*_3_/ITGB3/CD61 specific monoclonal antibody and anti-GAPDH as a housekeeping platelet protein. After washing, membranes were incubated with a secondary antibody coupled to HRP. Finally, bands were visualized using a ChemiDoc XRS+system and Image Lab software (Bio-Rad Laboratories, Inc. USA; Supporting Information Figure S1).The blots used in the figures are the original ones obtained by the system instrument and were not grouped or cropped from different parts of the same gel, or different gels or fields.

### 2.4. Statistical Analysis

To compare the expression of platelet surface molecules CD41, CD61, and activated *α*_IIb_*β*_3_ integrin as well as cell adhesion/spreading on different days, data were analyzed by the Kruskal–Wallis test with Dunn's multiple comparison tests. Mann–Whitney *U* test was also applied to compare abovementioned parameters between two groups (platelets treated with VAS2870 or NAC compared to control). Correlations were analyzed by Pearson's correlation coefficient. The *p*-values of less than 0.05 were considered to be significant, using the GraphPad Prism software (San Diego, CA).

## 3. Results

### 3.1. Storage-Dependent Cleavage of the Integrin *β*_3_ Chain in Platelet Concentrate

**Figure 1:**
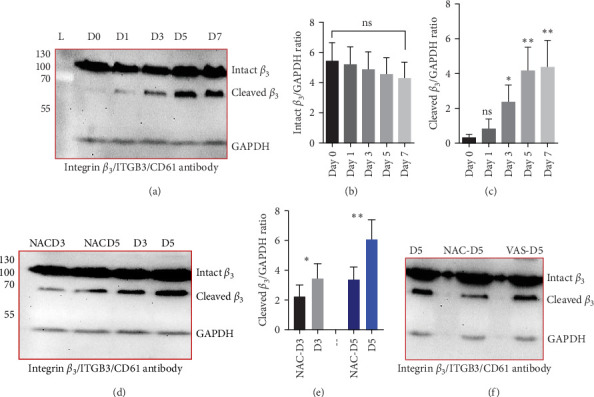
Integrin *β*_3_ chain cleavage in the presence/absence of NAC (1 mM) or VAS2870 (30 μM). The representative western blots show the levels of *β*_3_ cleavage in non-treated platelets (control) (A) and platelets treated with NAC (1 mM) (B). Graphs (D) and (E) demonstrate the levels of *β*_3_ chain expression and *β*_3_ cleavage during 7 days of storage, respectively. As shown in (F), 1 mM NAC significantly reduced the levels of *β*_3_ cleavage compared to the control in 3- and 5-day-stored platelets. However, VAS2870 had no significant effect on the cleavage of the integrin *β*_3_ chain in 5-day-stored platelets (C). ns, not significant; NAC, N-acetyl-L-cysteine. *⁣*^*∗*^*p* < 0.05, *⁣*^*∗∗*^*p* < 0.01, *n* = 10.

Figure 1A shows a representative western blot depicting expression of the *β*_3_ chain of integrin during storage, with a trend towards reduced expression during storage, albeit statistically insignificant (Figure 1B). In addition to this, an additional band identified by the same anti-*β*_3_ antibody was also detected migrating at a reduced molecular weight (MW) (with the size between 55 kDa and 70 kDa), with its expression inversely increasing during storage. The existence of such a smaller size of the *β*_3_ chain was consistent with a previous comprehensive study that used different types of anti-*β*_3_ antibodies to detect different patterns of calpain-dependent cleavage of the integrin, which also showed a similar cleavage pattern with the resulting chains of approximately 100 kDa and 67 kDa *β*_3_ [22]. These results suggest storage-dependent proteolytic cleavage of the *β*_3_ chain of integrin *α*_IIb_*β*_3_ (Figure 1A,C). Intriguingly, a gradual time-dependent increase in the smaller *β*_3_ band occurred along with a gradual decrease in the intensity of the major *β*_3_ band, suggesting storage-dependent cleavage of the *β*_3_ integrin chain by the presentation of two different sizes of *β*_3_ in the same gel.

### 3.2. The Effect of Reducing State on the Cleavage of *β*_3_ Chain of Integrin in Stored Platelets

Figure 1D shows a representative western blot of the *β*_3_ chain of integrin expression comparing the control platelet product with the NAC-treated one. As shown, treatment with NAC significantly reduced the cleavage of the *β*_3_ chain of integrin from day 3 of storage (Figure 1E). However, a comparison of NAC-treated platelets with VAS2870 (in 5 day stored platelets) showed that this irreversible NOX inhibitor had no significant effect on *β*_3_ integrin chain cleavage (Figure 1F). In comparing these two antioxidant agents, it should be noted that NAC acts as a general ROS scavenger that can reduce the total amount of intracellular free radicals produced by any source, whereas VAS2870, as an NOX inhibitor, only affects cytosolic ROS production. This is consistent with our previous study comparing ROS reduction using these two reducing agents, which confirmed a greater reducing effect of NAC compared to VAS2870 in stored platelets [36].

### 3.3. The Effect of Reducing State on the Surface Expression of *α*_IIb_*β*_3_ Integrin

**Figure 2:**
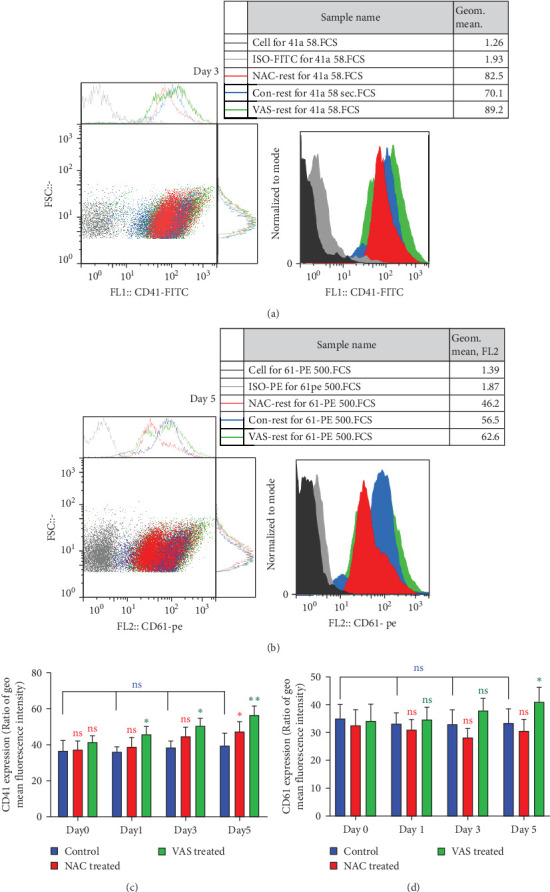
The surface expression of *α*_IIb_*β*_3_ integrin in the presence/absence of NAC (1 mM) or VAS2870 (30 μM). (A) and (B) show dotted plots and their corresponding histograms depict the levels of CD41 (*α*_II*b*_) and CD61 (*β*_3_) surface expression in platelets treated with either NAC or VAS2870 compared to non-treated platelets (control) obtained from 3- and 5-day stored PCs. Graphs (C) and (D), respectively, also show the effects of either NAC or VAS2870 treatments on the levels of CD41 (*α*_IIb_) and CD61 (*β*_3_) surface expression compared to control during 5 days of platelet storage. ns, not significant; NAC, N-acetyl-L-cysteine. *⁣*^*∗*^*p* < 0.05, *⁣*^*∗∗*^*p* < 0.01, *n* = 10.

As previously published and shown here, the expression of CD41 (*α*_IIb_) and CD61 (*β*_3_) does not change during platelet storage (Figure 2). As shown in Figure 2C, whereas treatment of platelets with VAS2870 significantly increased the CD41 expression from day 1 to day 5 of storage, NAC-treated platelets only increased its expression in 5 day stored platelets compared to the control. On the other hand, considering CD61 (*β*_3_), the data only showed increased levels of *β*_3_ expression in 5 day stored VAS2870-treated platelets compared to the control, whereas NAC-treated platelets did not show changes in its expression (Figure 2D). Figure 2A,B also show dot plots and their corresponding histograms for each assay on platelets obtained from days 3 and 5 of storage.

### 3.4. The Effect of Reducing State on the Activation Status of *α*_IIb_*β*_3_ Integrin

**Figure 3:**
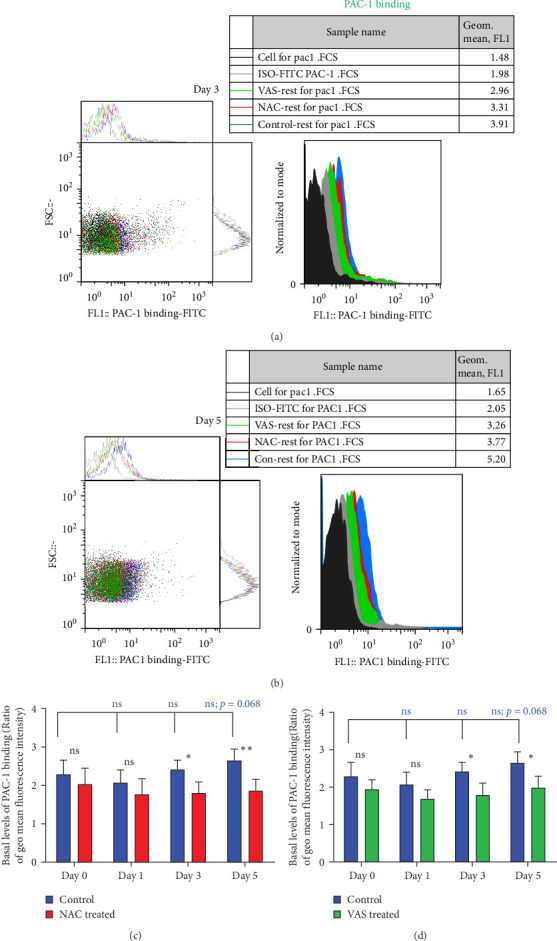
The level of PAC1 binding in the presence/absence of NAC (1 mM) or VAS2870 (30 μM). (A) and (B) show dotted plots and their corresponding histograms depict the levels of PAC1 binding in platelets treated with either NAC or VAS2870 compared to non-treated platelets (control) obtained from 3- and 5-day stored PCs. Bar charts (C) and (D), respectively, also show the effects of NAC and VAS2870 treatments on the basal level of PAC1 binding compared to control during 5 days of platelet storage. ns, not significant; NAC, N-acetyl-L-cysteine. *⁣*^*∗*^*p* < 0.05, *⁣*^*∗∗*^*p* < 0.01, *n* = 10.

At each stage, from preparation to storage, platelet products per se show different levels of inevitable activation. Therefore, for this study, for the first time, the basal levels of integrin activation in the absence of further stimulation with an agonist were evaluated by flow cytometry-based PAC-1 binding analysis for detection of activated *α*_IIb_*β*_3_. Studies that monitored conventional PAC-1 binding assays in response to weak agonists typically showed its gradual decrease during storage, indicating PSL-induced impairment of integrin functional activity. However, here assessment of basal levels of PAC-1 binding (in the absence of platelet stimuli) revealed a gradual increase in integrin activation but not significant (a trend significance; *p*=0.073) on day 0 of storage (PC preparation day) followed by partial declines on day one of storage and subsequent increases (also with a trend significance; *p*=0.068) on day 5 of storage (Figure 3C). This pattern is consistent with some previous studies that showed in a spontaneous experimental setting (without the use of an agonist), fibrinogen binding to platelets does not change during storage [37, 38]. As shown in Figure 3C,D, both NAC and VAS2870 treatment of platelets significantly reduced integrin activation from day 3 of storage with a more significant reduction observed by NAC in 5 day stored platelets. Given that platelet aggregation is regulated by the functional capacity of the *α*_IIb_*β*_3_ integrin, this finding is consistent with previous studies showing that NAC-treated platelets have impaired aggregation responses to various agonists [34]. Figure 3A,B also show dot plots and their corresponding histograms for each assay on platelets obtained from days 3 and 5 of storage.

### 3.5. The Effect of Reducing State on the Platelet Adhesion to Fibrinogen Matrix During Storage

**Figure 4:**
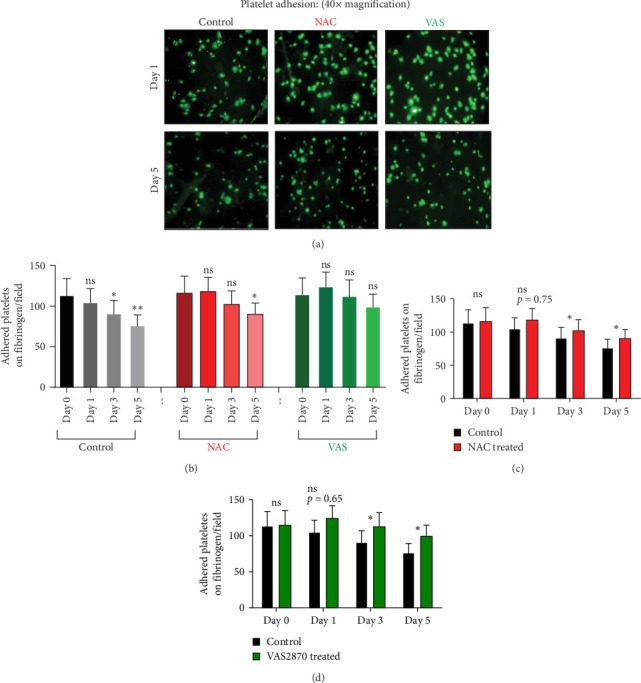
The levels of platelet adhesion to the fibrinogen matrix in the presence/absence of NAC (1 mM) or VAS2870 (30 μM). (A) Demonstrates the levels of platelet adhesion to the fibrinogen matrix in platelets treated with either NAC or VAS2870 and non-treated platelets (control) on days 1 and 5 of storage. Graph (B) shows the number of adhered platelets to the fibrinogen matrix in non-treated platelets and platelets treated with either NAC or VAS2870 during 5 days of storage. Bar charts (C) and (D), respectively, also illustrate the effects of NAC and VAS2870 treatments on the levels of platelet adhesion to the fibrinogen matrix versus non-treated platelets (control) during storage. ns, not significant; NAC, N-acetyl-L-cysteine. *⁣*^*∗*^*p* < 0.05, *⁣*^*∗∗*^*p* < 0.01, *n* = 10.

Figure 4A represents screenshots from microscopic fields (40× magnification) of platelets adhered to the fibrinogen matrix in either non-treated control or in platelet products treated with NAC/VAS2870. As shown by previous studies as well as here in Figure 4B, the number of adhered platelets to the fibrinogen matrix gradually decreases during storage with a significant decline starting from day 3. A relatively similar pattern was also shown in NAC-treated platelet products, while VAS2870-treated platelets did not show significant changes during storage (Figure 4B). In this regard, comparative studies also showed higher levels of platelet adhesion in both NAC- and VAS2870-treated stored platelets compared to non-treated controls with a significant increase observed from day 3 of storage in VAS2870/NAC-treated platelets (Figure 4C,D).

### 3.6. The Effect of Reducing Agents, NAC, and VAS2870 on the Platelet Spreading on Fibrinogen Matrix During Storage

We already described platelet adhesion/spreading to fibrinogen matrix as a reliable and sensitive marker of platelet functional activity during storage. In this regard, similar to PAC-1 binding assay while simple adhesion of platelet to fibrinogen matrix reflects aligation capacity of activated integrin, through further interaction of platelets with fibrinogen matrix, their shape change and spreading also provide more information about thecapacity of outside in signaling pathway induced by full activation and clustering of integrin *α*_IIb_*β*_3_. Platelet spreading, which occurs following cytoskeletal rearrangement and cell surface growth, unlike a simple attachment (similar to PAC-1 binding) or primary adhesion to fibrinogen, is a dynamic process that essentially requires a sufficient energy source [32]. Therefore, since platelet storage, especially in the long-term, is accompanied by metabolic stress, its effects on platelet spreading can be observed and assessed more tangibly. On the other hand, given the increased cleavage of the *β*_3_ integrin chain during storage and the importance of its cytoplasmic portion in outside in signaling [39], the spreading assay may also better reflect the functional activity of integrin [40]. This could be of special importance in the redox conditions induced by NAC treatment of platelets where this reducing agent was shown here to significantly reduce *β*_3_ cleavage during storage.

**Figure 5:**
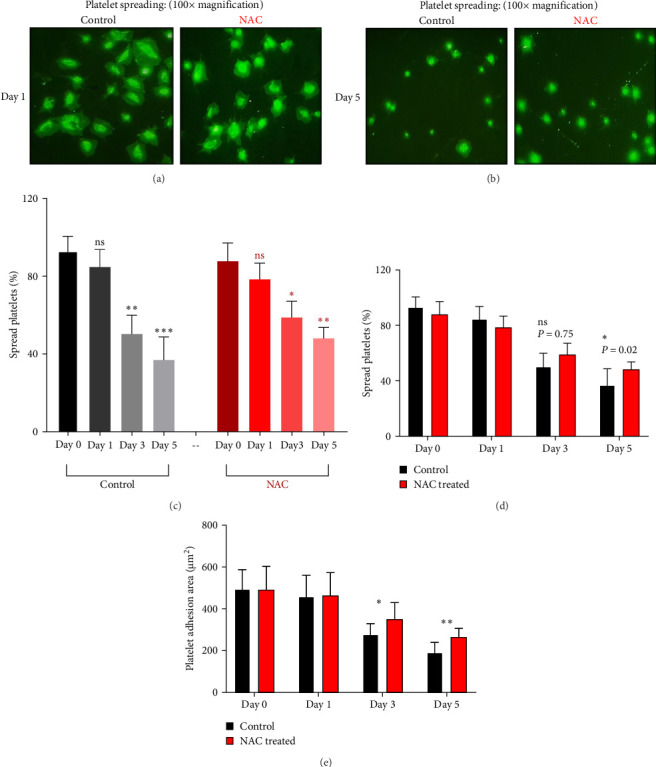
The levels of platelet spreading on the fibrinogen matrix in the presence/absence of NAC (1 mM). (A) and (B), respectively, illustrate microscopic fields (100× magnification) of platelet spreading on the fibrinogen matrix in non-treated platelets (control) versus NAC-treated platelet products on days 1 and 5 of storage. Graph (C) shows the percentage of spread platelets on the fibrinogen matrix in non-treated platelets (control) and platelets treated with NAC during 5 days of storage. As shown in (D), 1 mM NAC significantly improved the levels of platelet spreading on the fibrinogen matrix compared to non-treated (control) in 5 days stored platelets. Bar chart (E) shows the platelet adhesion area on the fibrinogen matrix in the presence of NAC versus non-treated (control) during 5 days of storage. ns, not significant; NAC, N-acetyl-L-cysteine. *⁣*^*∗*^*p* < 0.05, *⁣*^*∗∗*^, *p*  < 0.01, *⁣*^*∗∗∗*^*p* < 0.001, *n* = 10.

In this context, as shown by previous studies as well as here, the percentage of spread platelets on the fibrinogen matrix also gradually decreased during storage with a significant decline starting from day 3 (Figure 5). Similarly, while NAC-treated platelets also showed decreasing levels of spread platelets on the fibrinogen matrix during storage, in a comparative analysis, NAC treatment significantly improved platelet spreading from day 3 of storage (based on spreading/adhesion area; Figure 5).

**Figure 6:**
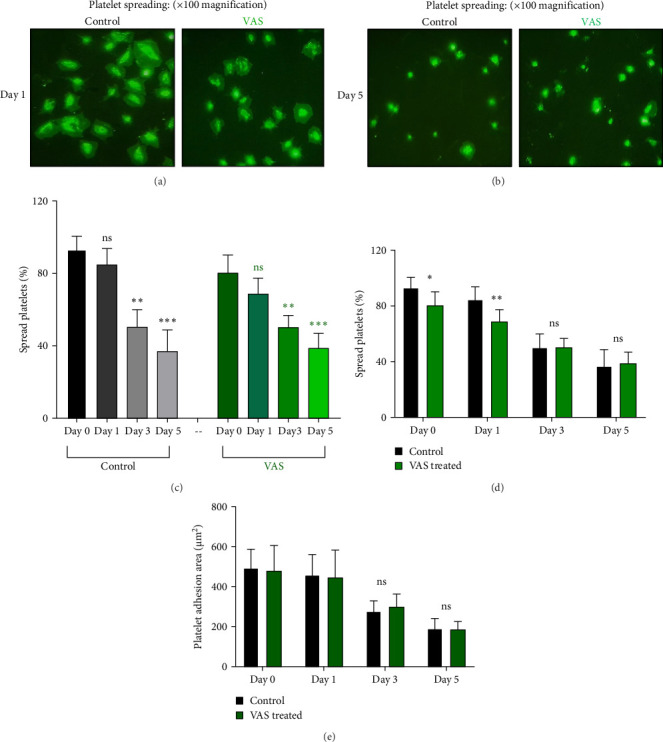
The levels of platelet spreading on the fibrinogen matrix in the presence/absence of VAS2870 (30 μM). (A) and (B), respectively, show microscopic fields (100× magnification) of platelet spreading on the fibrinogen matrix in non-treated platelets (control) versus VAS2870-treated platelet products on days 1 and 5 of storage. Graph (C) demonstrates the percentage of spread platelets on the fibrinogen matrix in non-treated platelets (control) and platelets treated with VAS2870 during 5 days of storage. As illustrated in (D), 30 μM VAS2870 significantly reduced the percentage of spread platelets on days 0 and 1 of storage compared to the non-treated (control). Bar chart (E) shows the platelet adhesion area on the fibrinogen matrix in the presence of VAS2870 versus non-treated (control) during 5 days of storage. ns, not significant. *⁣*^*∗*^*p* < 0.05, *⁣*^*∗∗*^*p* < 0.01, *⁣*^*∗∗∗*^*p* < 0.001, *n* = 10.

However, regardless of NAC improvement on platelet spreading, VAS2870 treatment showed quite different patterns. In general, similar to the control, VAS2870-treated platelets also showed decreasing levels of spreading on the fibrinogen matrix during storage (Figure 6A–C). However, VAS2870 treatment not only did not improve platelet spreading but it significantly reduced the percentage of spread platelets in earlier days of platelet storage (days 0–1; Figure 6D). Alternatively, with a more precise analysis that covers both adhesion and spreading events, VAS2870 treatment neither increased nor decreased platelet adhesion area on the fibrinogen matrix during storage (Figure 6E). This is in contrast to the improving effect of NAC treatment on platelet spreading (Figure 5D,E) which was also associated with the significant reduction of *β*_3_ cleavage during storage which is not also observed in VAS2870 treated platelets (Figure 1F).

### 3.7. The Significant Correlation of Platelet Adhesion and Spreading With the Levels of Smaller Chain *β*_3_ Integrin During Storage

**Figure 7:**
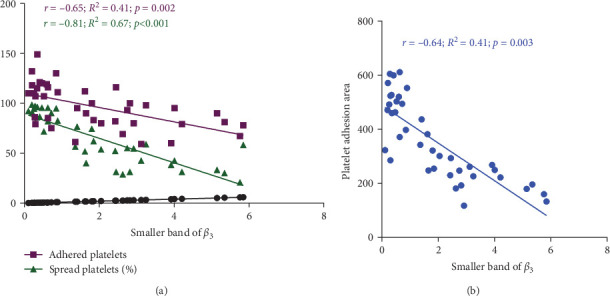
The correlation between platelet adhesion and spreading with the levels of the smaller band of *β*_3_ integrin in stored platelets. Graph (A) demonstrates a reverse correlation between both adhesion (*r* = −0.65; *p*=0.002) and spreading (*r* = −0.81; *p* < 0.001) with the smaller *β*_3_ integrin bands during platelet storage. Graph (B) also shows a reverse correlation between platelet adhesion area on fibrinogen and the smaller *β*_3_ integrin bands (*r* = −0.64; *p*=0.003) during storage.

Figure 7A demonstrates indirect correlations between platelet adhesion (*r* = −0.65; *R*^2^ = 0.41; *p*=0.002) and spreading (*r* = −0.81; *R*^2^ = 0.67; *p* < 0.001) on the fibrinogen matrix and the increasing expression of smaller *β*_3_ integrin bands during platelet storage. Alternatively, platelet adhesion area on fibrinogen was also significantly correlated with the expression of smaller *β*_3_ integrin bands (*r* = −0.64; *R*^2^ = 0.41; *p*=0.003) during platelet storage (Figure 7B).

## 4. Discussion

Given the fact that platelet storage is associated with both integrin activation and ROS generation, the study presented here tried to elucidate the link between storage-dependent oxidant stress and integrin functional capacity manifested by platelet adhesion and spreading to the fibrinogen matrix. In this regard, for the first time, this research showed a gradual increment in *β*_3_ cleavage during platelet storage leading to the increased intraplatelet expression levels of smaller *β*_3_ chain of *α*_IIb_*β*_3_ integrin. This was an important observation as was also in parallel with the decreasing adhesive capacity of platelets to fibrinogen in stored platelet products. At the next stage, the study also suggested that the scavenging of ROS by NAC could prevent *β*_3_ cleavage while enhancing platelet adhesion and spreading to fibrinogen in stored platelets.

Numerous studies so far have shown that the platelet activation state, especially under sustained calcium influx, can activate the intracellular proteolytic enzyme, calpain, that cleaves the *β*_3_ chain of *α*_IIb_*β*_3_ integrin affecting its functional activity in different ways [7, 23, 24]. Here, for the first time, western blot analysis (from nonreducing SDS-PAGE) by a specific antibody that recognizes residues close to the extracytoplasmic domain of *β*_3_ molecules, demonstrated thinner smaller bands of *β*_3_ (between 55 kDa and 100 kDa) associated with their original bands (~100 kDa) in stored platelets. Further analysis revealed an insignificant gradual decrease in the intensity of the major platelet *β*_3_ bands during PC storage, whereas the intensity of the smaller *β*_3_ bands increased inversely. Regardless of these patterns, the absence of smaller bands in fresh platelets (from 0 day stored PC) compared to those with the highest density at 7 days of storage (platelets with the most activation state) suggests that the smaller bands may represent integrin molecules that underwent calpain-dependent cleavage during storage. This is consistent with earlier studies, where using different types of specific antibodies against *β*_3_ truncated regions, integrin cleavage was shown under robust activating states with strong agonists [24]. However, to the best of our knowledge, this is the only report that shows smaller bands of *β*_3_ associated with the intact ones in stored platelets that reflecting activation-dependent changes of this molecule in platelet products.

So far, several lines of evidence have indicated how reducing state can decrease different patterns of platelet activation [41] including attenuation of the stored-dependent ectodomainshedding of adhesive receptors associated with their reduced functional activity [42]. However, whether reducing conditions also affect the expression, molecular conformation or functional activity of *α*_IIb_*β*_3_ integrin of platelets during storage has not been clearly elucidated yet. In this regards, data presented here for the first time showed that the treatment of platelet products (PRP-PCs) with 1 mM NAC could significantly decrease the levels of the second thinner and smaller *β*_3_ band of integrin in stored platelets, whereas surprisingly using VAS2870 as an irreversible specific inhibitor of cytosolic NOX with the safe concentration that was already effective in other studies [36, 43] did not show such an effect. This was a unique pattern as in our earlier studies on the shedding of platelet adhesive receptors both scavenger and inhibitor showed a similar reducing trend on receptor ectodomain cleavage [42]. Notably, given a previous study in which the optimized levels of these inhibitors were identified by the evaluation of their dose-dependent effects on platelet viability [36], treatment of platelet products with VAS2870 inhibitor more than 30 μM (which was used in this study) is also not recommended due to possible toxic effect on platelets. Therefore, we concluded that with its optimal concentration, VAS2870 as a specific NOX inhibitor cannot prevent *β*_3_ cleavage to the significant extent that was observed in NAC, the observation that may need further mechanistic investigation, especially with the use of other ROS inhibitors. In particular, it is important to note that calpain-dependent changes of *α*_IIb_*β*_3_ integrin usually occur under sustained calcium influx (in PS-positive platelets), which is generally associated with mitochondrial dysfunction [23, 44]. This is a condition in which platelets may show an extra ROS influx from mitochondrial sources that may not be inhibited by NOX inhibitors where it can be inactivated with NAC as a general ROS scavenger [45]. In other words, the observation of this pattern of *β*_3_ cleavage may indirectly be suggestive of the importance of the mitochondrial sources of ROS that secure enough elevation of oxidant stress required for the significant cleavage of *β*_3_, especially under the procoagulant state which mainly manifested in longer sorted platelets [46]. In a similar trend with a constant concentration of *β*_3_ levels detected in platelet lysate during storage, expression analysis of surface integrin molecules of CD61 (*β*_3_) and CD41 (*α*_II*b*_) in stored platelets did not detect any significant changes in platelet integrin expression during 5 days of storage, the observations that already shown in other studies [47, 48]. Further analysis also showed that treatment with VAS2870 enhanced this receptor expression, whereas NAC only increased CD41 expression in 5 days stored platelets compared to non-treated PCs. Whether the enhancement of integrin expression in reducing conditions reflects any changes in the activation status of these molecules or not was an important question that requires further investigation in the following stages of this research.

In this regard, the status of basal activation of integrin *α*_IIb_*β*_3_ was also evaluated here to identify the levels of inside out signaling that platelets receive during storage. Here, PAC-1 binding analysis demonstrated that, following a slight decrease on the first day of storage and in the absence of any experimental stimuli, the stored platelets per se showed increasing levels of integrin activation after the third day of storage (with a trend significance). The immediate post preparation increases in integrin activation levels of PCs appear to be due to the inevitable stressful condition of platelet isolation (e.g., during centrifugation stages), which is recovered by day 1 of storage under mild agitation. However, a second wave of increased integrin activation could reflect storage-dependent damage to platelets with upcoming activating inside out signals. Interestingly, further investigation here for the first time showed that both NAC and VAS2870 prevented integrin activation in stored platelets, with more significant inhibition achieved by NAC as a general scavenger of ROS that can attenuate the generated ROS from both cytosolic and mitochondrial sources [45, 49]. This is in line with other basic studies that showed how reducing status can attenuate platelet integrin activation state [50, 51], albeit those researches were not conducted on stored platelet products. The observed increasing PAC-1 binding is considered a sign of integrin priming and its increased affinity by inside out signaling during storage [52]. However, to evaluate the actual functional activity of integrin by outside in signaling further analysis is required. In addition, this would be very important to elucidate whether reducing state can improve the integrin-based platelet functionality in more dynamic assays.

To achieve this in the next stage of the study, platelet adhesion and its spreading capacity to the fibrinogen matrix were evaluated. For this purpose, washed platelets resuspended in Tyrode's buffer were let to adhere to immobilized fibrinogen, where the stored platelets gradually lost their adhesion and spreading capacity. While this is in line with other studies [32], the observation here that decreasing spreading was inversely correlated with increasing levels of the smaller fraction of *β*_3_ chains in our western blot analysis sounds to be an interesting finding. In this regard, studies have previously revealed that calpain-dependent cleavage of *β*_3_ integrin under sustained activation of platelets, such as that seen in PSLs, is associated with impaired spreading [24, 53]. Further analysis in the presence of ROS inhibitors showed increased platelet adhesion to the fibrinogen matrix in the presence of both NAC and VAS2870 compared to untreated control. The enhanced adhesion here was consistent with previous observations of higher integrin expression in the presence of reducing conditions, particularly given that VAS2870-treated platelets had the highest levels of CD41/CD61 expression associated with better adhesion, especially in longer stored platelets. However, it should be noted that due to the different functional nature of ROS inhibition by NAC and VAS2870, distinct inhibitory behaviors may also be exhibited by these inhibitors under different conditions. In this regard, NAC, as a scavenger, can reduce the amount of ROS already produced in platelets, while after removing NAC from the medium (e.g., by washing and resuspending platelets in a solution lacking it), these platelets can still continue to reversibly produce ROS. However, in contrast, VAS2870 is considered an irreversible inhibitor of NOX activity, which deprives platelets of the ability to produce ROS by this enzyme. This means that even after removal of VAS2870 from the medium, platelets previously treated with this irreversible inhibitor may no longer be capable of generating significant ROS following interaction with immobilized fibrinogen, whereas NAC-treated platelets after washing and resuspension in a new inhibitor/scavenger-free medium still have the mechanistic capacity to renovate ROS generation in reaction with any stimuli. This is a very important note for the interpretation of any adhesion-based assays, with this consideration that a physiologic concentration of ROS, as an important signaling molecule, is a prerequisite for the functional activity of platelets [29, 54], especially their spreading on reactive matrices. This is confirmed by several ex vivo studies that showed significant inhibition of platelet adhesion and thrombus formation on collagen and other reactive matrices in the presence of ROS inhibitors such as DPI and apocyanin [55, 56].

Given this introduction on the mechanistic note, while the study here showed increased platelet adhesion to fibrinogen matrix in the presence of both NAC and VAS2870 compared to untreated control, the improved platelet spreading was only achieved by NAC as a ROS scavenger with a reversible effect after platelet washing and resuspension. However, with regards to platelet spreading, not only VAS2870 inhibitor did not improve this functional pattern of platelets during storage but it also significantly reduced platelet spreading in earlier stages of platelet storage. Consistent with previous in vitro and in vivo studies, this observation may be justified by the fact that irreversible inhibition of ROS, whose threshold levels are normally required for platelet signaling, can permanently distrupt platelet activation potential, such that these inhibitory effects persist even after removal of the inhibitor by washing and resuspension steps [29, 43, 50]. On the other hand, NAC treatment not only enhanced platelet adhesion but also significantly increased platelet spreading on fibrinogen, especially in longer stored products. Here, while NAC successfully prevents PSL and especially integrin pre-activation during storage, it also well preserves the functional capacity of platelets for proper multistep adhesion and spreading on fibrinogen in our assay. This is due to the fact that, unlike VAS2870, the effect of NAC as a reversible ROS scavenger can be removed prior to the assay by washing and resuspension steps. In this condition, platelets still maintain their NOX activity to regenerate the necessary amounts of ROS required for appropriate signaling events leading to proper adhesion and spreading on fibrinogen [49, 54].

**Figure 8:**
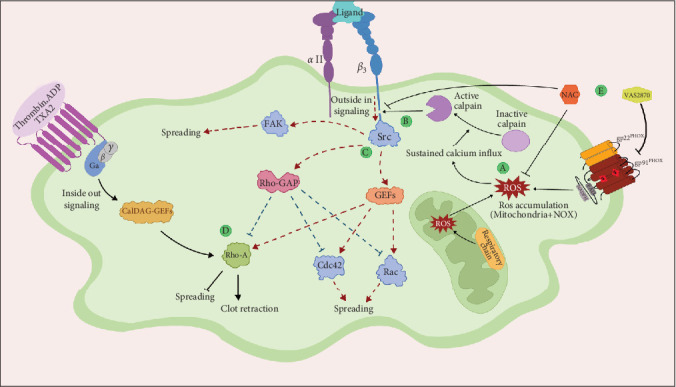
The mechanism behind downregulation of platelet spreading following calpain-dependent cleavage of the *β*_3_ integrin cytoplasmic tail. (A) ROS production via NOX and mitochondrial pathways following platelet activation is associated with sustained calcium influx. (B) An increase in intracellular calcium leads to the activation of calpain, which cleaves the binding site of *β*_3_ integrin by *c*-Src and results in its disassociation from the integrin tail. Src plays a substantial role in the phosphorylation and activation of signaling proteins, including focal adhesion kinase (FAK), RhoGAP, and GEFs. (C) GAPs and GEFs regulate the activation status of Rac1, Cdc42, and RhoA, which play an important role in platelet spreading. In addition, tyrosine phosphorylation of FAK can also affect platelet spreading. Following calpain activation and disassociation of *c*-Src, a significant defect occurs in these downstream pathways which leads to a decrease in platelet spreading. (D) The activation of RhoA-GEFs following ligand binding to different GPCRs increases the RhoA activity by induction of inside out signals. On the other hand, the inhibitory effect of Rho GAP on RhoA is relieved due to the disassociation of Src from the *β*_3_ integrin tail during calpain activity. Consequently, RhoA leads to the inhibition of platelet spreading and subsequently increases clot retraction (51). (E) The scavenging of ROS by NAC leads to a decrease in cleavage of *β*_3_ integrin tail as a result of the reduction of ROS accumulation, while VAS2870 as a specific NOX inhibitor cannot prevent possible cleavage of *β*_3_ (black lines, the occurrence of pathways; dashed lines, defects in these pathways). Cdc42, cell division control protein 42 homolog; FAK, focal adhesion kinase; GPCRs, G protein-coupled receptors; NOX, NAPDH oxidase; Rac1, ras-related C3 botulinum toxin substrate 1; RhoA, ras homolog family member A; RhoGAP, rho GTPase-activating proteins; RhoGEFs, rho guanine nucleotide exchange factors; ROS, reactive oxygen species. This is the original figure depicted by Dr.Ghasemzadeh's Lab.

However, in addition to the reversibility advantage of ROS inhibition by NAC, NAC-treated platelets here also showed a significant prevention of the production of smaller *β*_3_ integrin bands which may reflect *β*_3_ cleavage during platelet storage. Given the important role of the cytoplasmic tail of *β*_3_ in supporting outside in signals [24] and the fact that its calpain-dependent cleavage reduces platelet spreading (Figure 8), the prevention of *β*_3_ cleavage in the presence of NAC can be attributed to increased platelet spreading under such a reducing condition. Conversely, while VAS2870 treatment did not inhibit the generation of smaller bands of *β*_3_ integrin in our study, interestingly, it also failed to restore platelet spreading.

## 5. Conclusion

Now, given that NAC as a scavenger can reduce ROS originating from both NOX and mitochondrial sources, its achievement to rescue platelet spreading through inhibition of *β*_3_ cleavage, compared with VAS2870, which had no such effect, may highlight the fact that platelet spreading is primarily regulated by the cumulative effect of the mitochondrial source of ROS. Taken together, it seems that adding NAC to platelets can significantly preserve the optimal functioning capacity of integrin during storage, especially with the consideration that being a scavenger but not an irreversible inhibitor, the inhibitory effect of this reducing agent can be readily compensated after transfusion.

## Data Availability

The corresponding author can make available some datasets upon reasonable request.

## Supporting Information

Supporting Information Supporting Information Figure S1: Integrin *β*_3_ chain cleavage in the presence/absence of NAC (1 mM) or VAS2870 (30 μM). The representative western blots analysis and corresponding tables show the levels of *β*_3_ cleavage in non-treated platelets (control) (A) and platelets treated with NAC (1 mM) (B). (C) demonstrates the levels of *β*_3_ cleavage in NAC- or VAS2870-treated platelets in comparison with non-treated platelets (control) on day 5 of storage. NAC: N-acetyl-L-cysteine.
