# Supplementary material for: The Reactive Oxygen Species Scavenger N-Acetyl-L-Cysteine Reduces Storage-Dependent Decline in Integrin αIIbβ3-Mediated Platelet Function, Inhibiting Pre-Activation of Integrin and Its β3 Subunit Cleavage
Source: Oxid Med Cell Longev. 2025 Apr 21;2025:7499648. doi: 10.1155/omcl/7499648 (PMC12048192; doi:10.1155/omcl/7499648)
Supplement: Supporting Information — Figure S1: Integrin β3 chain cleavage in the presence/absence of NAC (1 mM) or VAS2870 (30 μM). The representative western blots analysis and corresponding tables show the levels of β3 cleavage in non-treated platelets (control) (A) and platelets treated with NAC (1 mM) (B). (C) demonstrates the levels of β3 cleavage in NAC- or VAS2870-treated platelets in comparison with non-treated platelets (control) on day 5 of storage. NAC: N-acetyl-L-cysteine. [file 7499648.f1.pptx]

## Slide 1
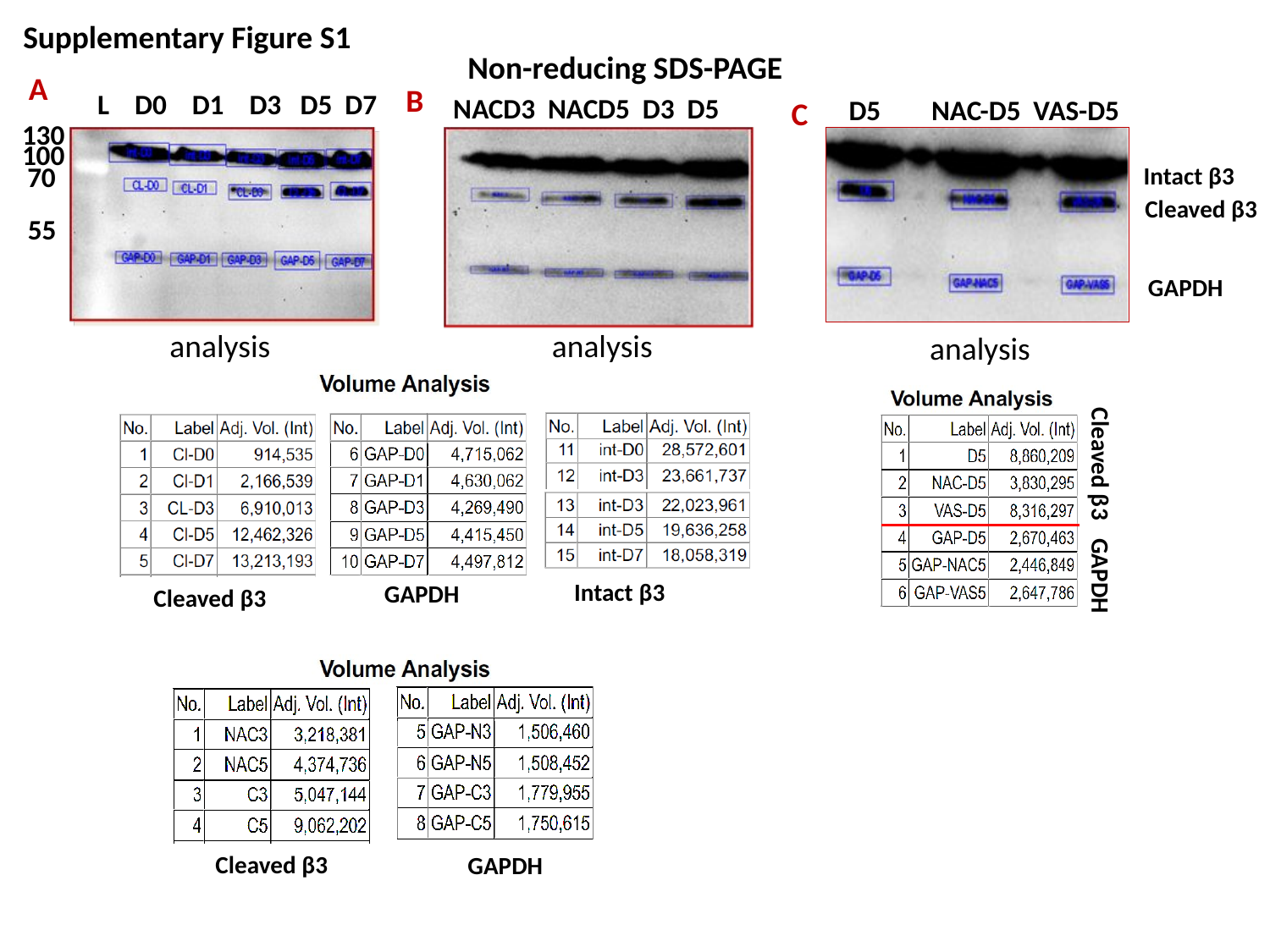

Supplementary Figure S1
Non-reducing SDS-PAGE
A
B
L D0 D1 D3 D5 D7
130
100
70
55
NACD3 NACD5 D3 D5
D5 NAC-D5 VAS-D5
C
Intact β3
Cleaved β3
GAPDH
analysis
analysis
analysis
Intact β3
GAPDH
Cleaved β3
Cleaved β3
GAPDH
Cleaved β3
GAPDH
